# Phylogeny and Niche Conservatism in North and Central American Triatomine Bugs (Hemiptera: Reduviidae: Triatominae), Vectors of Chagas' Disease

**DOI:** 10.1371/journal.pntd.0003266

**Published:** 2014-10-30

**Authors:** Carlos N. Ibarra-Cerdeña, Alejandro Zaldívar-Riverón, A. Townsend Peterson, Víctor Sánchez-Cordero, Janine M. Ramsey

**Affiliations:** 1 Departamento de Ecología Humana, Centro de Investigación y de Estudios Avanzados del IPN (Cinvestav), Unidad Mérida, Mérida, Yucatán, México; 2 Departamento de Zoología, Instituto de Biología, UNAM, México DF, México; 3 Biodiversity Institute, University of Kansas, Lawrence, Kansas, United States of America; 4 Centro Regional de Investigaciones en Salud Pública (CRISP), Instituto Nacional de Salud Pública (INSP), Tapachula, México; Universidad Autónoma de Yucatán, Mexico

## Abstract

The niche conservatism hypothesis states that related species diverge in niche characteristics at lower rates than expected, given their lineage divergence. Here we analyze whether niche conservatism is a common pattern among vector species (Hemiptera: Reduviidae: Triatominae) of *Trypanosoma cruzi* that inhabit North and Central America, a highly heterogeneous landmass in terms of environmental gradients. Mitochondrial and nuclear loci were used in a multi-locus phylogenetic framework to reconstruct phylogenetic relationships among species and estimate time of divergence of selected clades to draw biogeographic inferences. Then, we estimated similarity between the ecological niche of sister species and tested the niche conservatism hypothesis using our best estimate of phylogeny. *Triatoma* is not monophyletic. A primary clade with all North and Central American (NCA) triatomine species from the genera *Triatoma*, *Dipetalogaster*, and *Panstrongylus*, was consistently recovered. Nearctic species within the NCA clade (*T. p. protracta, T. r. rubida*) diverged during the Pliocene, whereas the Neotropical species (*T. phyllosoma, T. longipennis, T. dimidiata* complex) are estimated to have diverged more recently, during the Pleistocene. The hypothesis of niche conservatism could not be rejected for any of six sister species pairs. Niche similarity between sister species best fits a retention model. While this framework is used here to infer niche evolution, it has a direct impact on spatial vector dynamics driven by human population movements, expansion of transportation networks and climate change scenarios.

## Author Summary

Knowledge regarding the evolutionary history of insect vectors of pathogens is essential to design precise and appropriately integrated control strategies, since species' dispersal and invasive capacity are key components to prevent human-vector interaction. Given several well-known invasive or dispersal events of Triatominae, and their adaptive capacity to colonize modified habitats, the question arises whether niche conservatism (i.e. the limited capacity for niche differentiation within clades) across sister species could influence distribution patterns. Niche conservatism is herein analyzed across the highly heterogeneous landmass of North and Central America where most species complexes of the Triatominae are vectors of *Trypanosoma cruzi*. Since phylogenetic relationships among and within triatomine species complexes are still ill-defined, we first used a multi-locus phylogenetic analysis using mitochondrial and nuclear loci to validate relationships between species. Similarity of the ecological niche between six sister species' pairs was used to test the niche conservatism hypothesis. The results provide robust evidence of monophyly for North and Central America triatomine species. The hypothesis of niche conservatism was not rejected for any sister species pair, independently of niche width, distribution range, biogeographic affinity, or environmental heterogeneity; niche similarity correlated inversely with taxa divergence and according to a retention model.

## Introduction

Triatomine bugs (Reduviidae: Triatominae) are vectors of *Trypanosoma cruzi*, the etiologic agent of Chagas disease, which is the most common parasitic disease in the Americas [1]. Although *T. cruzi* transmission can occur without vector involvement (i.e. *T. cruzi* contamination through infected bug feces in human dermis or mucosae), the rate of other non-vector transmission cases such as congenital and oral transmission (i.e. involuntary ingestion of infected bugs, or blood or meat of infected mammals), blood transfusions, laboratory accidents and organ transplants [2]–[5] are generally lower in vector endemic regions [6], [7]. Domiciliated vectors are considered the primary exposure hazard for *T. cruzi* transmission to humans [8]–[10] and triatomine ecology and evolutionary history, and their relationship with the geographic expression of its fundamental niche, is a primary determinant [11]–[13].

The Triatominae, commonly named assassin bugs, is a subfamily that belongs to the large family Reduviidae. The family includes 6600 species classified into 21 subfamilies [14]. It is the least diverse among reduviid subfamilies, having approximately140 species grouped into 11 genera [15]; [14], [16]. Yet, new species continue to be described in recent years [17] and a new revision of the taxonomy of this group is increasingly necessary [18]. Triatomine bugs are characterized by their obligate haematophagy [19], which is considered to have evolved once or twice [11], [16]. The bloodsucking habit and its associated morphological adaptations have been highly influential in taxonomic definition of the subfamily, however, recent morphological and genetic studies are challenging the current classification of Triatominae, as well as clarifying some phylogenetic relationships at different taxonomic levels. For instance, some studies on the *Triatoma dimidiata* complex [20]–[22], or the *T. brasiliensis* [23] subcomplex have highlighted the need for a generic revision within the Triatomini tribe.

Triatomine species are mainly distributed in the New World, except for seven species that occur in Asia. In the Americas, they occupy tropical, subtropical and temperate areas approximately between the 40th parallels [24]. Despite wide environmental variation across North America [25], geographic and environmental ranges of triatomine species tend to be similar [12]. This similarity could be explained by niche conservatism, in which diversification in ecological niche dimensions has been limited [26].

In vector-borne disease systems, vector niche conservatism patterns could influence a particular suite of processes in pathogen dynamics, for instance, to which areas dispersal could occur, and/or with what hosts the vector interacts [27]. Different macro-evolutionary models have been used to explain niche conservatism patterns and divergence rates such as drift, phylogenetic inertia, niche filling/shifting, evolutionary rate, and niche retention [28]. In particular, the niche retention model proposes lower expected divergence, which would be the result of stabilizing selection, or evolutionary constraints such as those imposed by developmental, physiological, or population-level genetic factors [29]–[32].

Ecological niche modeling (ENM) is a correlative framework that uses the associations between aspects of climate and environmental characteristics, and known species' occurrences, to define sets of conditions under which species are likely to maintain viable populations. The ENM can be projected onto geographic space, thereby depicting the potential area for each species' distribution [33]. This approach has been widely applied to study several aspects of the geographic distribution of Chagas disease vectors [34].

Warren et al. [35] developed a statistical framework to take advantage of niche model outputs so that the method could be used to test for niche conservatism: testing whether two niches are identical or whether two niches are more similar than expected at random. Niche similarity or divergence between species are not *per se* proof of niche conservatism, or a lack thereof [35]–[37]. Since niche similarity between species could be a result of niche conservatism or of niche convergence [38], specific evidence for phylogenetic relationships are crucial for niche comparison interpretation. Additionally, several considerations are essential to correctly interpret niche conservatism in sibling species: (1) compared taxa should be the most likely sister species, (2) the sister species should be located in a region of probable historical accessibility of both species [39], [40], and (3) niche predictor variables must be selected based on biological significance and absence of statistical redundancy [41], [42]. All three design criteria need to be addressed in order to avoid ambiguity as a result of inconsistency in sibling species selection criteria [43], multi-dimensionality of dataset predictors (which causes overfitting) [41], and failure to integrate geographically explicit potential dispersal areas [36].

In the present study, we test whether niche conservatism is a common pattern for the genus *Triatoma* of North and Central America (NCA) by using combined phylogenetic and biogeographic approaches. Since phylogenetic relationships among triatomines are still a matter of debate [14]–[16], [44], [45], we first reconstructed relationships among New World triatomines, and then analyzed whether phylogenetic patterns were associated with geography, thereby indicating that bioclimatic niches evolve in a continuous and historically-accessible landmass. The hypothesis of niche conservatism between phylogenetically validated *Triatoma* sister species from NCA was analyzed using niche similarity.

## Materials and Methods

### Taxa and molecular sequences used in analyses

Triatomine species from North (Mexico and United States) and Central America (partial territory of Guatemala and Belize) located within the North American tectonic plate, and for which at least three of the six gene markers examined were available in GenBank (see below), were included in this study. Sequences from 53 triatomine species from seven genera (*Dipetalogaster, Eratyrus, Paratriatoma, Panstrongylus, Mepraia, Rhodnius*, and *Triatoma*), representing 40% of all described species of the subfamily for the entire American continent and the primary Chagas disease vectors, were analyzed. *Triatoma* species were classified following Lent and Wygodzinsky' taxonomic revision [46] except for *T. dimidiata*, which was divided into three terminal taxa according to evidence suggesting that this is a complex of cryptic species [20]–[22], [47], [48]. We have named the analyzed taxa according to Bargues et al. [20] and Monteiro et al. [22] which names *T. dimidiata* group 1a for the populations inhabiting the coast of Chiapas as well as Guatemala, Honduras and Nicaragua; *T. dimidiata* group 2 for those inhabiting Gulf and central Mexico, and *T. dimidiata* group 3 for those from the Yucatan Peninsula. In this study we analyze 19 of 31 species representing 60% of the *Triatoma* diversity along the latitudinal and ecological spectrum of the North and Central America region [12]. Two species belonging to the reduviid subfamily Reduviinae (*Zelurus petax* and *Reduvius personatus*) were used as outgroups to root all trees [11], [49].

### Phylogenetic analyses

Previously published (GenBank) sequences belonging to four mitochondrial (mt) and two nuclear gene markers were used to carry out a concatenated phylogenetic analysis. Mt markers included a fragment covering most of cytochrome oxidase I (COI) (1494 bp), 682 bp of cytochrome b (cyt b), 375 bp of 12S ribosomal RNA (rRNA), and 604 bp of 16S rRNA genes. Nuclear markers were ∼1919 bp of the 18S rRNA and ∼620 bp of the 28S rRNA genes.

Gene sequences were aligned manually, and ambiguously aligned regions for the rRNA genes were excised using Gillespie's et al. [50] secondary structure model approach. Our concatenated data set included 87% of species for 16S, 63% for 12S, 52% for cyt b, 48% for COI, 37% for 18S and 30% for 28S. A list of the examined species, genes analyzed and GenBank accession numbers is included in Table S1. The genes included for each of the phylogenetic analyses performed are listed in Table S2. References for published sequences used in the above phylogenetic analyses are listed in Table S3.

The concatenated data set was analyzed with the partitioned Bayesian method implemented in MrBayes version 3.2.1 [51]. The analysis consisted of independent runs of four parallel chains and 20 million generations each, using uniform priors, and sampling trees every 1000 generations. Protein-coding genes were divided into three partitions based on codon positions, whereas each rRNA marker was considered as a single partition. JMODELTEST version 2 [52] was used to select the evolutionary model appropriate for each partition following the Akaike Information Criterion (AIC). Stationarity was determined to have occurred before 1 million generations, although we used a conservative approach and deleted the first 5000 sampled trees. The remaining trees were used to reconstruct a tree with posterior probabilities (PP) of clades, considering that clades with PP≥0.95 were significantly supported [53].

Separate Bayesian and maximum likelihood analyses (ML) including the 19 species from the NCA clade (see below) were carried out for the cyt b and ITS markers (Table S2). The Bayesian analyses were conducted using the same parameters described above. The Tamura-Nei model using MEGA version 5 [54] was used for the ML analyses based on a bootstrap consensus tree inferred from 1000 replicates. Branches corresponding to partitions reproduced in <50% of bootstrap replicates were collapsed. Additionally, a discrete Gamma distribution was used to model evolutionary rate differences among sites (4 categories +G; cyt *b* parameter = 0.54; ITS2 parameter = 0.57). The rate variation model for cyt *b* allowed for some sites to be invariable ([+I], 49.9% sites).

### Tests of alternative topologies

We tested our preferred hypothesis of phylogeny against three alternative topologies using Bayes factor comparisons, which were calculated from estimates of marginal likelihoods using the Stepping Stone (SS) sampling approach, which uses importance sampling to estimate each ratio in a series (the “stepping stones”), bridging posterior and prior distributions [55], implemented in MrBayes version 3.2 [51]. The three alternative topologies analyzed were: (1) a monophyletic *Triatoma*, (2) *Triatoma* species from NCA constrained to be monophyletic, and (3) the combined species from *Triatoma*, *D. maximus*, and *P. hirsuta* from NCA constrained to be monophyletic. The SS model estimates were obtained by running 10 million generations, followed by 50 steps with 1000 samples within each step, and eliminating the first 25% of samples from each step. Two independent runs were performed for each dataset, one for a constrained and one for a non-constrained topology. The arithmetic difference of the Bayes factors for the two runs in log units is the criterion used to evaluate the null hypothesis (monophyly). A log difference in the range of 3–5 is typically considered strong evidence in favor of a model, whereas a log difference above five is considered very strong evidence [56].

### Divergence times

We estimated divergence time of clades using a relaxed molecular clock approach with BEAST version 1.7.4 [57]. We excluded *R. personatus* from this analysis in order to have a basal node separating the remaining Triatominae from *Z. petax*. The G+I+Γ model was used for this analysis, considering each gene marker as a single partition. The analysis was run for 20 million generations and sampling trees, every 1000 generations. The first 10 million generations were discarded, and the remaining trees were used to build a maximum clade credibility tree with TreeAnnotator version 1.7.4 (part of BEAST 1.7.4).

The most basal node indicating separation between Reduviinae and Triatominae in our BEAST topology was calibrated to have a normal prior distribution of 52.89 my with 4.5 my standard deviation. This calibration was defined based on the divergence time estimate recovered by Hwang and Weirauch [11] for the most recent common ancestor (MRCA) of the above two subfamilies. The MRCA of the Triatomini clade was calibrated to have a normal prior distribution 30.0 my with 1 my standard deviation, based on the age assigned to a *Triatoma* fossil from Dominican amber [58].

### Sister species data points and definition of the background area for each pair

We identified six pairs of sister species belonging to our phylogenetically recovered NCA *Triatoma* clade for niche conservatism analysis. Only one sister species pair (*T. gerstaeckeri* –*T. mexicana*), was defined based on data from the single locus analysis of both cytb and ITS2, while the rest were supported with the multilocus analysis. Georeferenced occurrence locations reported elsewhere were used to build ENM models [12], [27], [59] (see Ibarra-Cerdena et al. [12]) for a description of occurrence dataset sources). Niche models were constructed for members of each species for pairs, using 188 data points for *T. barberi*, 477 for *T. dimidiata* group 2, 42 for *T. dimidiata* group 1a, 235 for *T. gerstaeckeri*, 115 for *T. longipennis*, 42 for *T. mazzottii*, 265 for *T. mexicana*, 22 for *T. nitida*, 33 for *T. phyllosoma*, 154 for *T. p. protracta*, 44 for *T. recurva*, and 42 for *T. rubida* (Table S4). The “M” region [60] or “background area” in the terminology of Warren et al. [35], was defined for each species by plotting the species' occurrence points on the map of terrestrial eco-regions of the world [61]. We used that map to outline the ecoregions where datapoints of species were located, then we used that contour to create a spatial subset of the bioclimatic (WorldClim) and topographic (Hydro-1k) rasters which were used for model calibration and randomization tests, as has been recommended elsewhere for “M” delineation [40], [62].

### Ecological niche modeling

Ecological niche models (ENM) were produced using the Genetic Algorithm for Rule-set Prediction (GARP; [63]. GARP is an evolutionary-computing software package available in openModeller Desktop version 1.1.0 (http://openmodeller.sourceforge.net/). To take advantage of the random-walk nature of the GARP algorithm, we developed 100 replicate models of each species' ecological niche and chose a ‘best subset’ of the 100 models based on optimal combinations of error statistics, which is also implemented in openModeller [64]. To choose best model subsets, we (1) eliminated all models that had omission error >5% based on independent extrinsic test points, (2) calculated the median area predicted present among these low-omission points, (3) identified the 10 models closest to the overall median area predicted, and (4) summed these ‘best subsets’ models. Binary models were obtained from the 10 best models by using a minimum presence threshold criterion in which a binary projection (presence-absence) is a result of the percentage of model agreement that predicts the presence of all data points.

The WorldClim “bioclimatic” data [65] include 19 variables that summarize aspects of climate relevant to species distributional ecology. We analyzed correlations among these variables and excluded variables from highly correlated variable pairs (r>0.75). Variables most easily interpretable in terms of species physiological tolerances were retained. Nine variables were retained in model development (annual mean temperature, maximum temperature of warmest month, minimum temperature of coldest month, annual temperature range, temperature seasonality, annual precipitation, precipitation of wettest and driest months, and precipitation seasonality), as well as four topographic variables from the Hydro-1K dataset (elevation, slope, aspect, compound topographic index; Earth Resources Observations and Science-http://eros.usgs.gov/products/elevation/gtopo30/gtopo30.html; last accessed Dec, 2011), at the 0.01° resolution (approx. 1 km).

### Testing niche similarity

We applied a randomization test of background similarity to evaluate the niche conservatism hypothesis. To test if sister species pairs had more similar ecological niche than expected by chance, we conducted a background similarity test using ENMTools version 1.3 [66]. Briefly, a similarity metric Schoener's D [35], was calculated from ENM generated in MaxEnt [67], [68] with the “minimum presence training” threshold tool activated. The modeling parameters were random test percentage of 75% and a maximum number of 500 iterations. A null distribution for these distances was calculated based on comparison of models for each species' occurrence with models generated using random occurrence points within the M of its sister species. The null hypothesis (species similarity not different than expected) can be rejected when empirical similarity values are lower (niche divergence) than the 100 random similarity replicates (95% confidence interval, P<0.05), based on a one-tail test.

## Results

### Phylogenetic relationships

**Figure 1:**
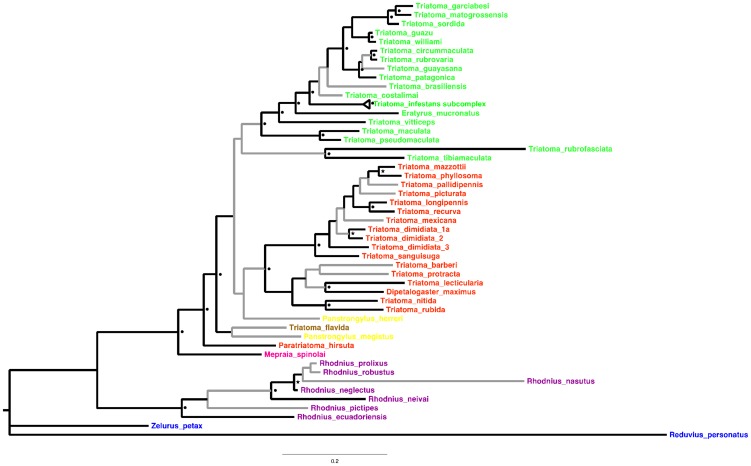
Bayesian phylogram derived from a multilocus analysis of the Triatominae subfamily, includes *Zelurus petax* and *Reduvius personatus* from the Reduviinae as outgroup (in blue). The geographic range for all species modeled in the genus *Triatoma* is also shown (NCA species are shown in red, South American species in green and from the Antilles in brown). *Panstrongylus* species are shown in yellow, *Mepraia* in pink and *Rhodnius* species are in purple. Branch color indicates PP<0.8 in gray and ≥0.8 in black. Black circles indicate PP≥0.95<; black stars PP≥0.9<0.95.

The Bayesian phylogram derived from the concatenated analysis of the cyt *b*, COI, 12S, 16S, 18S and 28S gene markers is shown in Figure 1. The phylogram contains a considerable number of significantly supported clades (23 out of 49 clades), with three additional clades also having significant PP values (0.9≤PP≤0.94).

The tribes Triatomini and Rhodniini each appeared significantly supported as monophyletic (both with PP = 0.96). Species of *Triatoma* were nested in a significantly supported clade (PP = 0.96) together with two species of *Panstrongylus* (*P. herreri* and *P. megistus*), *D. maximus*, and *E. mucronatus*. This predominately-*Triatoma* group contains two clades (although not significantly supported), one exclusively composed of South American species, and the other of NCA species. Sister species from the NCA clade (Table 1; Figures S1 and S2) were supported by PP values≥0.90, except for *T. p. protracta*-*T. barberi* (PP = 0.56). This latter sister species pair was, however, significantly supported (PP = 1; BTP = 99) using the ITS2 Bayesian and ML topologies (Table 1; Figure S2). The separate cyt *b* and ITS2 ML analyses (Figures S1 and S2) also significantly supported *T. mexicana* and *T. gerstaeckeri* as sister species, as did the ITS2 analysis (PP = 0.96).

**Table 1:** Comparative phylogenetic analysis of sister species belonging to the NCA species complexes that were recovered from the Bayesian multilocus and single gene locus analyses.

Sister species	Multilocus	Cytb		ITS2	
	Bayesian	Bayesian	ML	Bayesian	ML
T. barberi-T. p. protracta	**✓**	NS	NS	✓***	✓***
T. nitida-T. rubida	✓***	✓***	**✓**	✓***	**✓** *
T. mexicana-T. gerstaeckeri	ND	**✓**	**✓**	✓**	**✓**
T. longipennis-T. recurva	✓***	✓***	**✓**	NS	NS
T. mazzottii-T. phyllosoma	**✓** *	NS	**✓**	✓***	✓**
T. dimidiata 1a-T. dimidiata 2	✓**	✓***	**✓**	✓***	✓**

(ML = maximum likelihood).

ND: No Data.

NS: Not Supported.

*Significance for Bayesian: *≥0.85<0.95, **≥0.95<0.99, ***≥0.99, for ML(%): *≥85<95, **≥95<99, ***≥99.

The three Bayes factors comparisons performed with the SS approach strongly supported the relationships derived from the Bayesian concatenated analysis (Figure 1), indicating that *Triatoma*, as well as the NCA species of *Triatoma*, are not monophyletic (monophyletic vs non-monophyletic *Triatoma* group = −28556.75 and −28475.20, respectively; monophyletic vs non-monophyletic NCA *Triatoma* group = −28563.94 and −28411.77, respectively; monophyletic vs non-monophyletic NCA triatomines = −28399.01 and −28653.88, respectively).

### Divergence time estimates

**Figure 2:**
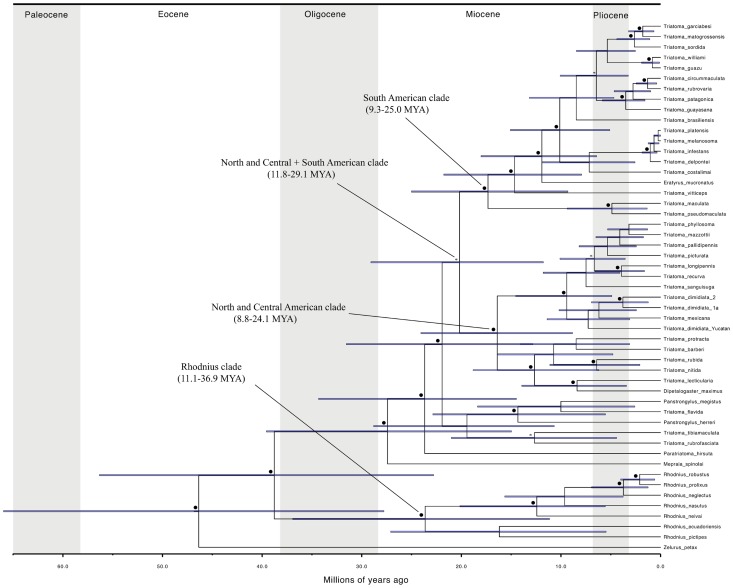
Divergence time estimates for Triatominae clades. Asterisks and black circles above branches indicate clades supported by PP of 0.9–0.94 and ≥0.95, respectively.

Divergence time estimates between the *Triatoma* species of NCA and South America are on the order of 14.1–22 my. Separation of the Central American clade (*Panstrongylus* spp. and the *rubrofasciata* complex) and that from North America (including Mexico) was estimated to have a similar age to the previous (13–20 my). Divergence between Nearctic (*T. p. protracta* and *T. r. rubida*) and Neotropical (*T. phyllosoma* and *T. dimidiata*) species was dated to have occurred during the Miocene (10–16 my). Speciation events of the Neotropical species occurred principally during the Pleistocene, in contrast to the Pliocene for the Nearctic species (Figure 2).

### Sister species ENM

**Figure 3:**
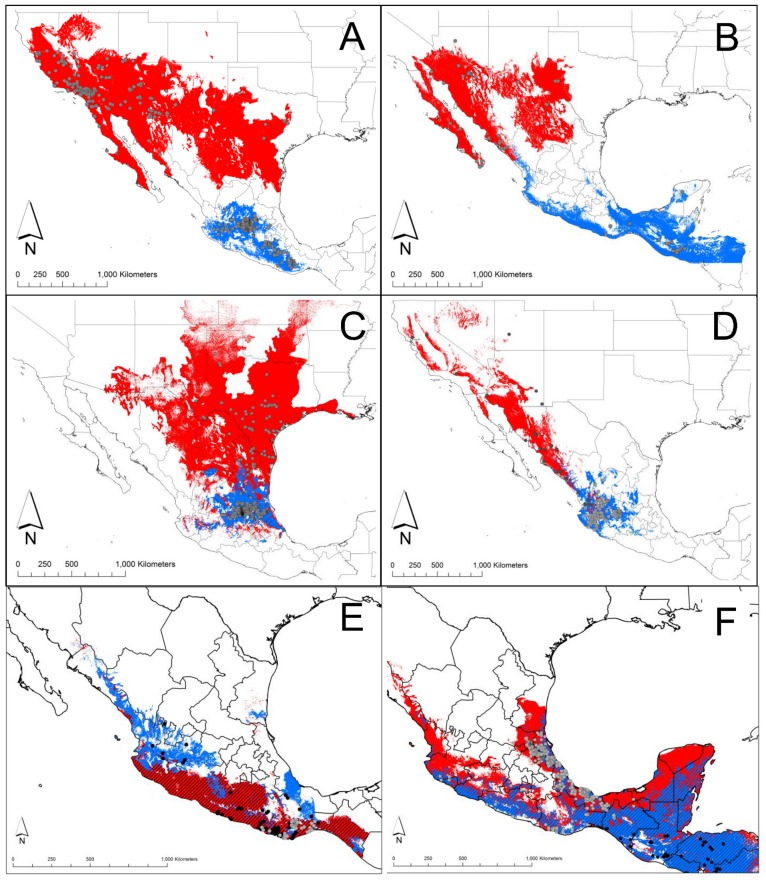
Ecological niche models for sister species pairs. A: *Triatoma p. protracta* (red) and *T. barberi*; B: *T. rubida* (red) and *T. nitida* (blue); C: *T. gerstaeckeri* (red) and *T. mexicana* (blue); D: *T. recurva* (red) and *T. longipennis* (blue); E: *T. phyllosoma* (red) and *T. mazzottii* (blue); F: *T. dimidiata* group 2 (red) and *T. dimidiata* group1a (blue). Grey dots and squares represent the collection sites for each species. Diagonal lines in E and F indicate the overlapping niche range between the sister pairs.

The ENM for all NCA *Triatoma* species covers most of the regional territory of Guatemala, Belize, El Salvador, Nicaragua, Honduras, Mexico and the southern United States (Figure 3). There was no range overlap between sister species of inter-biogeographical regions (i.e. the Neotropical *T. barberi* and the Neartic *T. p. protracta*), in contrast to the extensive range overlap in species pairs within the Neotropical region (*T. mazzottii*-*T. phyllosoma* and *T. dimidiata* groups 1a and 2; Figure 3E–F). The broadest potential distribution range was *T. p. protracta* in the US and Mexico, almost crossing the complete continental longitudinal gradient, while its sister species, *T. barberi*, spans both Nearctic and Neotropical regions, covering the highlands of the Transvolcanic Belt (Figure 3A). In general, sister species have allopatric potential distribution along a north/south latitudinal pattern (Figure 3A, 3B, 3C, 3D), although sub-tropical species pairs' potential distributions are partially sympatric (Figure 3E–F).

### Niche similarity tests

**Figure 4:**
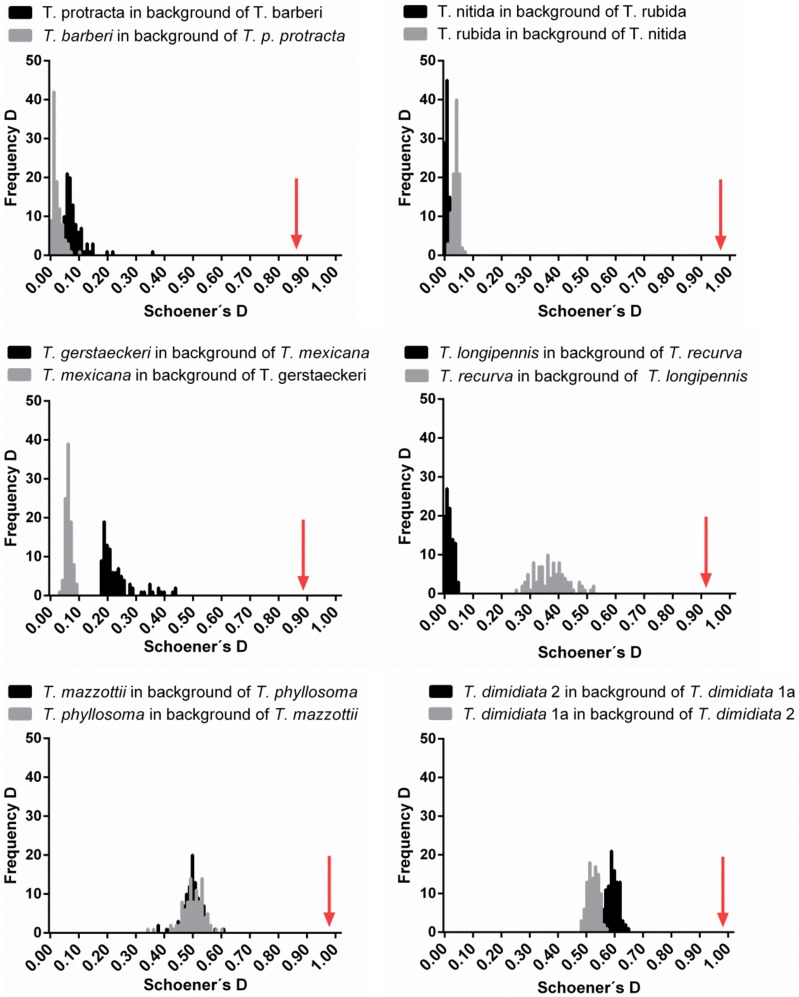
Ecological niche similarity tests between sister species pairs of NCA triatomines. Observed similarity between niches is indicated with the arrows, while bars indicate the null distribution of ecological niche distance generated randomly. Schoeners' D ranges from 0 (complete different ENM), to 1 (identical ENM). In all cases, the observed similarities were higher that their respective null distribution for random niche models.

The geographic distribution of all NCA *Triatoma* sister species niche was more similar between pairs than expected by chance (P<0.01). The observed niche similarity among sister pairs ranged from 0.88 to 0.99 (for Schoener's D metric; Figure 4). Niche similarity occurred despite high background divergence (the range of similarity values calculated from random niche models generated from the background area of each pair shown by the bars in Figure 4; the more the distance between the observed similarity and the mean of random similarities, the higher the contrast between the background areas of the two sister species), as demonstrated with the example of the average of 0.05 for the random “D” of the *T. p. protracta*-*T. barberi* sister pair (constructed with *T. barberi* points randomly generated in the background region of *T. p. protracta*).

Temperature seasonality (bio4 in WorldClim nomenclature) was one of the most important bioclimatic variables shaping the niche of 9 out of the 12 species. This variable was also the most important between three out of six sister pairs (*T. dimidiata* group 2 and *T. dimidiata* group 1a, *T. mazzottii* and *T. phyllosoma*, and *T. r. rubida* and *T. nitida*), having similar patterns of predicted suitability response curves. Other important variables were annual temperature range (bio7), minimum temperature of coldest month (bio 7), and precipitation of the wettest month (bio13). Conversely, topographic variables such as altitude or the topographic index were less conserved particularly between allopatric species (i.e. *T. barberi* and *T. p. protracta*, and *T. r. rubida* and *T. nitida*).

## Discussion

### Evolutionary history of New World and NCA triatomines

In our phylogenetic analyses, *Triatoma* is consistently recovered as not monophyletic with respect to at least three genera, which is supported by the Bayes factor tests. The results strongly support a main clade composed of NCA species of *Triatoma*, which also includes the monospecific *Dipetalogaster*, and at least those examined species of *Panstrongylus*. *Eratyrus* is deeply nested in the South American clade. *Paratriatoma* was either recovered within, or as sister, to the NCA clade in the MrBayes and BEAST analyses, respectively, and thus its placement remains uncertain. Our phylogenetic estimates also support previous studies based on single [44] and multi-locus genetic markers [23], [45], indicating a sister group relationship between the NCA and the South American *Triatoma* clades. In the most recent and comprehensive multilocus phylogenetic analysis of Reduviidae, the subfamily was paraphyletic with respect to the Reduviine genus *Opisthacidius*, although only a small sample of triatomine species was analyzed (less than 10% of the species described for the subfamily) [11]. Despite the use of an incomplete matrix of sequences to infer phylogenetic relationships in Triatominae, our results are highly compatible with recent phylogenetic reconstructions based on multilocus analyses [18], highlighting the ability of our Bayesian approach to extract the phylogenetic information contained in the molecular markers used. A number of recent empirical and simulation studies, have consistently demonstrated that even with a high proportion of missing data (up to 75%), this approach has a high accuracy in the reconstruction of phylogenetic relationships [69]–[73].

The MRCA of the NCA and South American *Triatoma* clades diverged approximately 14.1–22 my ago. Hypsa et al. [44], had proposed a Central American and/or Great Antilles origin for the Triatominae. A Central American origin for the Triatominae seems more likely, due to the presence in this region of many extant triatomine species from the genera *Eratyrus, Cavernicola, Panstrongylus, Triatoma* and the presence of *Opisthacidius*, recovered by Hwang and Weirauch [11] as the a sister group of Triatominae. Our divergence time estimates suggest a dispersal of Triatominae from Central to South America with a subsequent species radiation of the group in the latter region, which could have initiated after the connection of the Isthmus of Panama during the early Oligocene and Miocene [74].

The *T. protracta*, *T. rubida* and *T. lecticularia* complexes and the monotypic genera *Dipetalogaster* and *Paratriatoma*, a group of species inhabiting mainly the mid and southern area of the Nearctic region, diverged during the Pliocene, when major climate oscillations occurred [75], [76]. Moreover, most Neotropical NCA species (*phyllosoma* and *dimidiata* complexes) diverged during the Pleistocene. Several speciation events for Neotropical triatomines have been proposed before this period for South American triatomines [77], [78]. Our findings indicate that there were at least two speciation periods for the sister species that we tested for niche conservatism: (1) late Miocene-Early Pliocene (>5 my) for *T. p. protracta*-*T. barberi*, and *T. nitida*-*T. rubida*, and (2) late Pliocene-Pleistocene (<5 my) for *T. phyllosoma*-*T. mazzottii*, *T. longipennis*-*T. recurva*, and *T. dimidiata* group 2-*T.dimidiata* group 1a. None of the species from the NCA clade are significantly distributed beyond this region, and no species outside of the clade has a significant portion of its range within the NCA region.

### Niche conservatism within NCA triatoma

This study analyzes niche conservatism between sister species by first using a phylogenetic criterion to identify sister species. We have reconstructed phylogenetic relationships and estimated divergence times using a concatenated dataset, which consisted of six gene markers with differing degrees of missing data. Previous studies using both empirical and simulated data have shown that the accuracy of phylogenetic analyses is not influenced by the inclusion of missing data, but rather due to the amount of phylogenetic signal contained in the dataset examined [71], [72].

None of the niche conservatism hypotheses that were independently tested in six sister species from the NCA region was rejected. This niche conservatism occurs across the different species complexes of *Triatoma* from NCA. We observed a gradient in range size, distribution region, range overlap, and niche breadth in NCA triatomines. However, despite variations, niche conservatism was observed even for species' pairs with highly divergent background environments and allopatric distributions (*T. p. protracta* and *T. barberi* or *T. r. rubida* and *T. nitida*). Under the evolutionary species concept, retention of niche characteristics in highly divergent environments could promote speciation, since dispersal between geographic ranges is unlikely [79]. In contrast, the extended niche overlapping between Neotropical species pairs, suggests that sympatry is likely, if broad-scale dispersal occurs (i.e. driven by human movements). Previous studies regarding the *dimidiata* complex have used biased datasets to simply unite occurrence points and draw polygons in order to project species distributions over geographic space, demonstrating parapatric ranges [20], [22], or a minor measure of sympatry [80]. Ecological niche models have proved to be better tools to depict more reasonable range maps with ample application in vector-borne distribution assessments [34]. This robust and validated method demonstrates a large region of range overlapping that more realistically represents the current patterns of *dimidiata* complex distributions, as validated by more complete sampling and genetic analyses (Pech-May et al., *unpublished results*).

Here we tested whether niche conservatism is a general pattern for NCA triatomines regardless of the species complex to which they belong (i.e. *protracta*, *rubida*, *phyllosoma*, *dimidiata*, etc). We did not find evidence for ecological speciation, since niche similarity among the sister species was always higher than that of the background tests. Allopatric species often inhabit different environmental conditions and landscapes. Hence, the fact that their niches are not identical does not prove a lack of niche conservatism [35], [37]. Conversely, if the niche of two closely related species is more similar than expected despite non-overlapping ranges, this indicates that these retain niche traits, potentially due to a phylogenetic effect over-riding the spatial impact (i.e. the influence of dispersal and recent evolution in niche similarity) [81]. Phylogenetic effects (i.e. the influence of phylogenetic history on niche similarity) are assumed to limit the evolution of environmental tolerances that shape a species' distribution [28]. Lower tolerances to extreme climatic conditions were the most conserved niche attributes of NCA triatomines. Other studies have found that temperature and seasonality are the most important climatic determinants of triatomine species richness patterns in the American continent [82]. The implications of these findings will require further analysis, in light of deep phylogenetic niche conservatism in NCA triatomines [38].

### Implications of niche conservatism in spatial epidemiology

Niche conservatism in vectors with public health importance has important implications for disease transmission ecology [83]. Niche conservatism of disease vectors could impose constraints on adaptive responses to cope with climate change [84] or natural or anthropogenic dispersal (i.e. the growth of transportation networks and inter-locality human movement or migration) [85], [86]. Since NCA triatomines are both sylvatic and synanthropic [12], [59], [87], [88], they are prone to suffer involuntary translocations due to human migration. A well-documented case of this dispersal process was the Central American and southern Mexico invasion of *Rhodnius prolixus* [89]. This species succeeded in sub-tropical forest areas in southern Mexico and CA, although it was never collected in conserved habitats, possibly because niche conservatism prevented the species from establishing outside its ecological requirements. Recent distribution shifts for NCA triatomines have been recorded based on extra-range collections for *T. dimidiata* and sylvatic *T. nitida* and *T. mazzottii* [59], [88], [90]. The latter two records are inside the ecological range of each species, as predicted using niche conservatism analysis, but outside reported distribution areas.

Niche conservatism analysis contextualized in evolutionary history has provided a robust perspective for the geographic patterns observed in NCA triatomines. Accordingly, knowledge of the evolutionary trends in niche evolution can help to identify areas where vector control can be easily implemented for faster results (i.e. in sink habitats where low niche suitability prevents long term population establishment), while programing more intensive control campaigns in areas where high niche suitability guarantees appropriate climate for population expansion (source habitats). This knowledge can also help to generate dynamic risk maps that integrate environmental changes and the probability for shifts of disease vector species distribution ranges.

## Supporting Information

Figure S1 Bayesian and ML phylograms derived from the cyt *b* dataset for North and Central American Triatominae, using *P. megistus* as outgroup.
(TIF)

Figure S2 Bayesian and ML phylograms derived from the ITS2 dataset for North and Central American Triatominae using, *P. megistus* as outgroup.
(TIF)

Table S1 DNA sequences examined in this study, exact size (bp) for each marker and collection site of specimens.
(PDF)

Table S2 GenBank accession numbers of the sequences examined in this study.
(XLSX)

Table S3 References for the DNA sequences used in this study.
(PDF)

Table S4 Geographic coordinates of Triatomine species used in niche modeling.
(XLSX)
