# Supplementary figures and images for: Phylogeny and Niche Conservatism in North and Central American Triatomine Bugs (Hemiptera: Reduviidae: Triatominae), Vectors of Chagas' Disease
Source: PLoS Negl Trop Dis. 2014 Oct 30;8(10):e3266. doi: 10.1371/journal.pntd.0003266 (PMC4214621; doi:10.1371/journal.pntd.0003266)

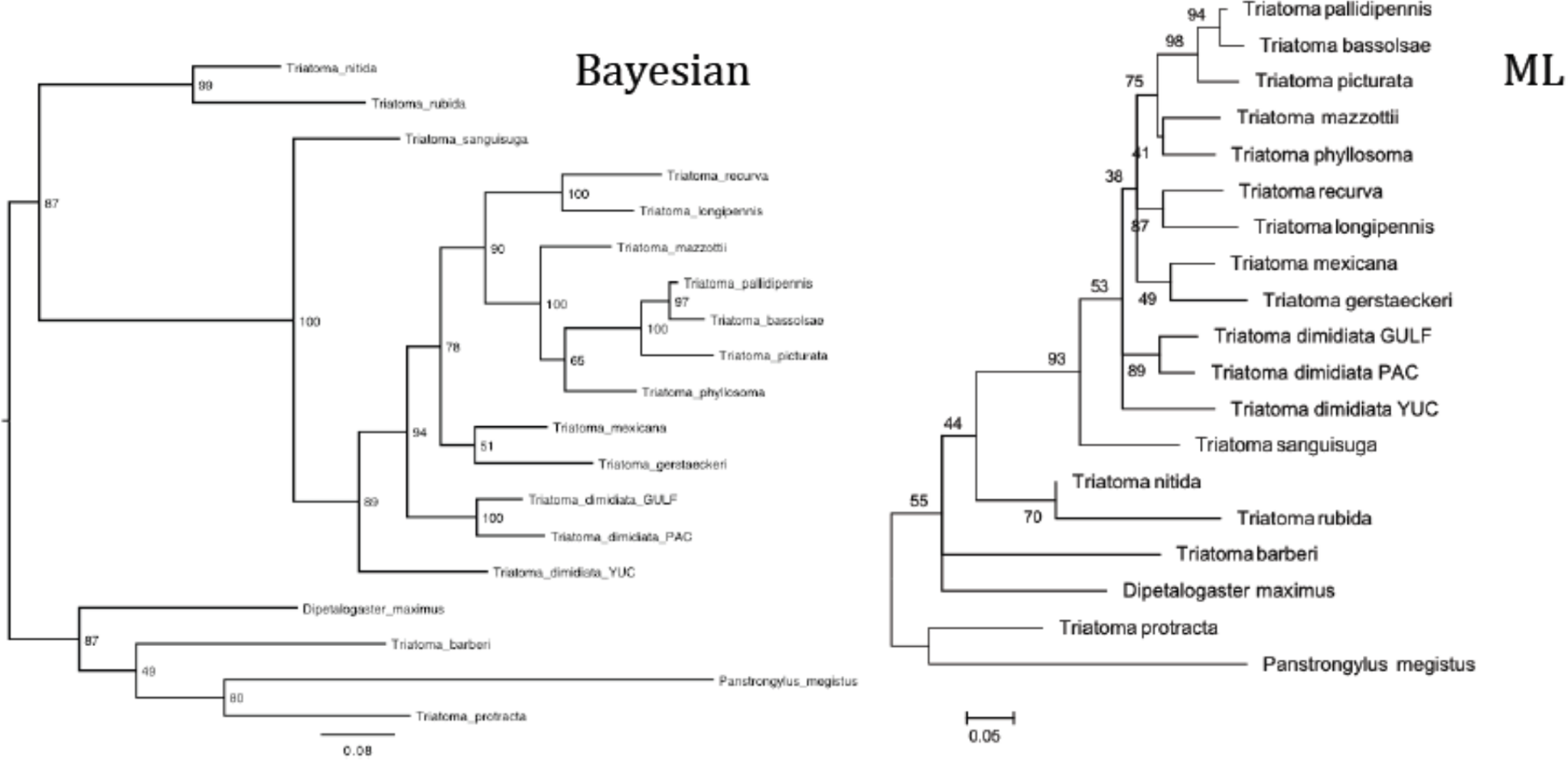

Supplement: Figure S1 — Bayesian and ML phylograms derived from the cyt b dataset for North and Central American Triatominae, using P. megistus as outgroup. (TIF) [file pntd.0003266.s001.tif]

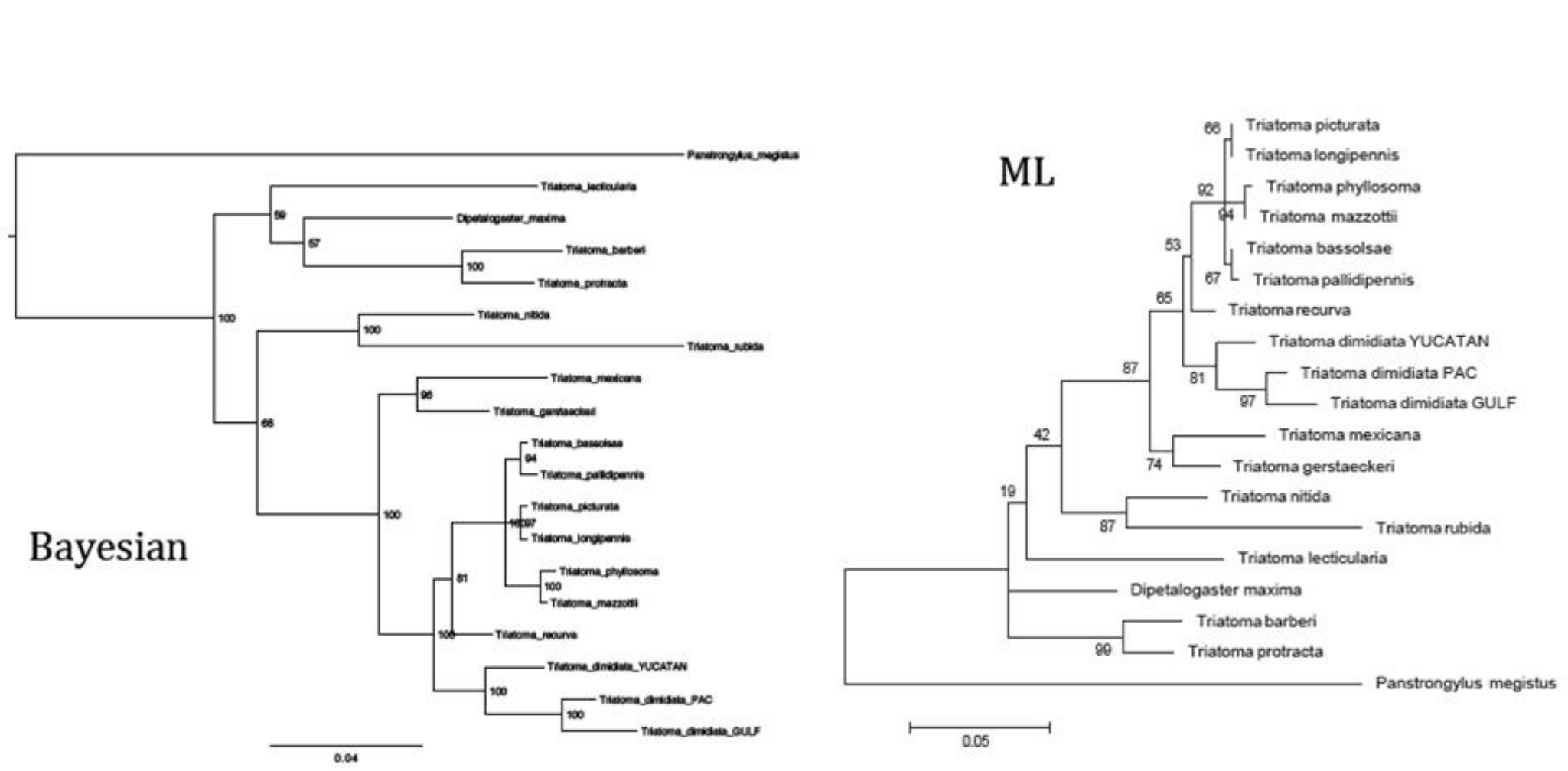

Supplement: Figure S2 — Bayesian and ML phylograms derived from the ITS2 dataset for North and Central American Triatominae using, P. megistus as outgroup. (TIF) [file pntd.0003266.s002.tif]
