# Supplementary material for: Phylogeny and Niche Conservatism in North and Central American Triatomine Bugs (Hemiptera: Reduviidae: Triatominae), Vectors of Chagas' Disease
Source: PLoS Negl Trop Dis. 2014 Oct 30;8(10):e3266. doi: 10.1371/journal.pntd.0003266 (PMC4214621; doi:10.1371/journal.pntd.0003266)
Supplement: Table S1 — DNA sequences examined in this study, exact size (bp) for each marker and collection site of specimens. (PDF) [file pntd.0003266.s003.pdf]

Species	Gene	Accesion Number	bp	Locality	Country
Triatoma barberi	cytb	AY830137	313	Oaxaca	Mexico
	18S	AJ421958	1913	Morelos	Mexico
	ITS2	AJ293590	470	Oaxaca	Mexico
Triatoma bassolsae	ITS2	AM286724	490	Puebla	Mexico
Triatoma brasiliensis	cytb	FJ594113	371	Piaui	Brazil
	COI	AF021186	1447	N/A	Brazil
	12S	AF021187	342	N/A	Brazil
	16S	AF021185	507	N/A	Brazil
	18S	AJ421957	1913	Ceara	Brazil
	28S	GQ853395	636	Rio de Janeiro	Brazil
Triatoma circummaculata	COI	AF021191	552	N/A	N/A
	12S	AF021190	342	N/A	N/A
	16S	AF021189	507	N/A	N/A
Triatoma costalimai	12S	AY185820	342	Goias	Brazil
	16S	AY185834	507	Goias	Brazil
Triatoma delpontei	cytb	HQ333241	388	Chaco	Bolivia
	COI	FJ439768	661	Santiago del Estero	Argentina
	12S	AF324510	342	N/A	N/A
	16S	AF324520	507	N/A	N/A
Triatoma dimidiata Pac	cytb	AY062157	665	Santa Rosa	Guatemala
	16S	AY062139	499	Tegucigalpa	Honduras
	ITS2	AM286693	497	Jutiapa	Guatemala
Triatoma dimidiata Gulf	cytb	AY062149	665	Veracruz	Mexico
	16S	AY062132	499	Veracruz	Mexico
	18S	AJ243328	1913	Oaxaca	Mexico
	ITS2	AM286714	494	Hidalgo	Mexico
Triatoma dimidiata Yuc	cytb	FJ197159	665	Yucatan	Mexico
	16S	AY062147	499	Yucatan	Mexico
	ITS2	FJ197152	494	Cayo	Belize
Triatoma flavida	16S	AY035451	510	N/A	N/A
	18S	AJ421959	1913	Guanacahibes	Cuba
Triatoma garciabesi	COI	EF451041	661	N/A	Bolivia
	12S	AY185821	342	Rivadavia	Argentina
	16S	AY185835	507	Rivadavia	Argentina
Triatoma gerstaeckeri	ITS2	AM286734	483	San Luis Potosi	Mexico
Triatoma guayasana	COI	AF021193	1447	N/A	N/A
	12S	AF021196	342	N/A	N/A
	16S	AF021194	507	N/A	N/A
Triatoma guazu	12S	AY185822	342	N/A	N/A
	16S	AY035457	506	N/A	N/A
Triatoma infestans	cytb	AY702024	441	N/A	Bolivia
	COI	FJ811848	601	Chaco	Argentina
	12S	AY226895	371	N/A	Argentina
	16S	EU143699	507	Buenos Aires	Argentina
	18S	Y18750	1914	N/A	N/A
	28S	GQ853397	648	N/A	South America

Triatoma lecticularia	cytb	AY859414	313	Nuevo Leon	Mexico
	12S	AY185823	341	Oklahoma	USA
	16S	AY185837	510	Oklahoma	USA
	28S	GQ853378	666	N/A	Brazil
	ITS2	AY860407	551	Nuevo Leon	Mexico
Triatoma longipennis	cytb	DQ198815	682	Zacatecas	Mexico
	COI	DQ198804	636	Zacatecas	Mexico
	18S	AJ243331	1913	Jalisco	Mexico
	ITS2	AJ286883	470	Zacatecas	Mexico
Triatomamaculata	COI	AF449139	1431	N/A	N/A
	12S	AF324512	342	N/A	N/A
	16S	AY035465	506	N/A	N/A
Triatoma matogrossensis	12S	AF394521	341	N/A	Brazil
	16S	AY035454	507	N/A	Brazil
	28S	GQ853398	636	Rio de Janeiro	Brazil
Triatoma mazzotti	cytb	DQ198816	682	Oaxaca	Mexico
	COI	DQ198805	636	Oaxaca	Mexico
	12S	AF324514	341	N/A	Mexico
	16S	AF324527	509	N/A	Mexico
	18S	AJ243333	1913	Oaxaca	Mexico
	ITS2	AJ286885	468	Oaxaca	Mexico
Triatoma melanosoma	12S	AY185824	342	Misiones	Argentina
	16S	AY185838	507	Misiones	Argentina
Triatoma mexicana	cytb	DQ118976	312	Hidalgo	Mexico
	COI	DQ198807	636	Guanajuato	Mexico
	ITS2	AM286728	492	Hidalgo	Mexico
Triatoma nitida	cytb	AF045723	399	N/A	N/A
	16S	AF045702	371	N/A	N/A
	ITS2	AM286733	490	El Progreso	Guatemala
Triatoma pallidipennis	cytb	EU790632	313	Jalisco	Mexico
	12S	AY185825	341	Different localities	Mexico
	16S	AY185839	510	Different localities	Mexico
	18S	AJ243330	1913	Morelos	Mexico
	ITS2	AM286729	491	Morelos	Mexico
Triatomapatagonica	12S	AF324515	343	N/A	N/A
	16S	AY035464	506	N/A	N/A
Triatoma phyllosoma	cytb	DQ198818	682	Oaxaca	Mexico
	COI	DQ198806	636	Oaxaca	Mexico
	18S	AJ243329	1913	Oaxaca	Mexico
	ITS2	HQ185172	469	Oaxaca	Mexico
Triatoma picturata	cytb	DQ198817	682	Jalisco	Mexico
	12S	AY185826	340	Nayarit	Mexico
	16S	AY185840	509	Nayarit	Mexico
	18S	AJ243332	1913	Jalisco	Mexico
	ITS2	AJ286884	470	Jalisco	Mexico
Triatoma platensis	COI	AF021202	1447	Tosquea	Argentina
	12S	AY226891	371	Tosquea	Argentina

	16S	AF021201	507	Tosquea	Argentina
	28S	GQ853400	651	N/A	N/A
Triatoma proctata	Cytb	AF045727	399	N/A	N/A
	12S	AF324516	341	N/A	N/A
	16S	AY035444	507	California	USA
	18S	FJ230520	955	California	USA
	28S	GQ853383	621	California	USA
	ITS2	JQ282715	402	Chihuahua	Mexico
Triatoma seudomaculata	12S	AY185827	342	Different localities	Brasil
	16S	AY185841	506	Different localities	Brasil
Triatoma recurva	Cytb	DQ198813	682	Arizona	USA
	COI	DQ198803	636	Sonora	Mexico
	16S	FJ230417	554	Sonora	Mexico
	18S	FJ230496	955	Sonora	Mexico
	28S	FJ230577	660	Sonora	Mexico
	ITS2	JQ282717	400	Sonora	Mexico
Triatoma rubida	Cytb	DQ198808	682	Arizona	USA
	COI	DQ198800	636	N/A	Mexico
	12S	AY185828	341	N/A	N/A
	16S	AY185842	507	N/A	N/A
	28S	GQ853391	679	N/A	N/A
	ITS2	AM286735	516	Nayarit	Mexico
Triatoma rubrofasciata	COI	GQ869655	530	N/A	N/A
	16S	AY035468	508	N/A	N/A
	18S	AJ421960	1913	Chalcatiznigo	Mexico
	28S	GQ853371	642	N/A	N/A
Triatoma rubrovaria	Cytb	GQ398005	359	Rio Grande do Sul	Brasil
	COI	AF021206	1447	N/A	N/A
	12S	AF021207	342	N/A	N/A
	16S	GQ423626	267	Rio Grande do Sul	Brasil
Triatoma sanguisuga	Cytb	AF045725	399	N/A	USA
	16S	AF045696	374	N/A	USA
	28S	GQ853392	658	N/A	USA
Triatoma sordida	Cytb	AF045730	399	N/A	Brasil
	COI	AF021216	1447	N/A	Brasil
	12S	AF021211	340	N/A	Brasil
	16S	AF021212	508	N/A	Brasil
	18S	AJ421956	1913	MatoGrosso	Brasil
Triatoma tibiamaculata	12s	AY185829	341	N/A	Brasil
	16S	AY185843	508	N/A	Brasil
Triatoma vitticeps	COI	AF021219	1447	N/A	Brasil
	12S	AF021217	342	N/A	Brasil
	16S	AF021218	507	N/A	Brasil
Triatoma williami	12S	AY185830	342	N/A	N/A
	16S	AY185844	506	N/A	Brasil
Eratyrus mucronatus	COI	AF449140	1431	N/A	N/A
	12S	AY185817	341	N/A	N/A

	16S	AY035450	507	N/A	N/A
	18S	AJ421953	1913	N/A	N/A
Dipetalogaster maximus	Cytb	AF045728	399	BCS	Mexico
	12S	AF394524	339	BCS	Mexico
	16S	AY035442	508	BCS	Mexico
	18S	AJ243334	1913	BCS	Mexico
	ITS2	AJ286887	475	BCS	Mexico
Mepraia spinolai	COI	GQ336894	631	Caleta Punta Sierra	Chile
	12S	AF324507	341	N/A	Chile
	16S	AF324518	508	N/A	Chile
	18S	AJ421961	1913	N/A	Chile
Panstrongylus megistus	Cytb	AF045722	399	Boca da mata	Brasil
	COI	AF021182	1447	N/A	Brasil
	12S	AF021180	341	N/A	Brasil
	16S	AF045701	371	N/A	Brasil
	18S	AJ243336	1913	Belo horizonte	Brasil
	28S	GQ853381	665	N/A	N/A
	ITS2	AJ306542	600	Minas Gerais	Brasil
Panstrongylus herreri	COI	AF449141	1431	N/A	N/A
	12S	AY185818	342	N/A	Peru
	16S	AY185833	506	N/A	Peru
Paratriatoma hirsuta	16S	FJ230443	552	California	USA
	18S	FJ230521	955	California	USA
	28S	FJ230604	657	California	USA
Reduvius Personatus	COI	AY318911	809	N/A	South Africa
	12S	AF394517	348	N/A	N/A
Rhodnius ecuadoriensis	cytb	AF045715	399	N/A	N/A
	16S	AF028746	285	N/A	N/A
Rhodnius nasutus	12S	AF394520	342	N/A	N/A
	16S	AF028749	248	N/A	N/A
	28S	AF435856	621	Piaui	Brazil
Rhodnius neglectus	cytb	AF045716	399	N/A	Brazil
	16S	EU822951	315	Guaira	Brazil
Rhodnius nevai	COI	AF449137	1431	N/A	N/A
	16S	AY035441	508	N/A	N/A
Rhodnius pictipes	cytb	FJ887792	645	N/A	N/A
	COI	AF449136	1431	N/A	N/A
	16S	EU827215	312	Jacunda	Brazil
Rhodnius prolixus	cytb	AF421339	663	Orica	Honduras
	COI	AF449138	1431	N/A	N/A
	12S	AF394519	344	N/A	N/A
	16S	EU827206	316	Madre de Dios	Peru
	18S	AJ421962	1918	Rio de Janeiro	Brazil
	28S	AF435860	633	N/A	N/A
Rhodnius robustus	cytb	FJ887793	645	N/A	N/A
	12S	AF394518	340	N/A	N/A
	16S	EU827206	316	Madre de Dios	Peru

	28S	AF435861	633	Trujillo	Venezuela
Zelurus petax	16S	FJ230416	552	N/A	Ecuador
	28S	FJ230576	657	N/A	Ecuador
