# Supplementary material for: Phylogeny and Niche Conservatism in North and Central American Triatomine Bugs (Hemiptera: Reduviidae: Triatominae), Vectors of Chagas' Disease
Source: PLoS Negl Trop Dis. 2014 Oct 30;8(10):e3266. doi: 10.1371/journal.pntd.0003266 (PMC4214621; doi:10.1371/journal.pntd.0003266)
Supplement: Table S3 — References for the DNA sequences used in this study. (PDF) [file pntd.0003266.s005.pdf]

1 Martinez,F.H., Villalobos,G.C., Cevallos,A.M., Torre Pde,L., Laclette,J.P., Alejandre-Aguilar,R. and Espinoza,B. 2006. Taxonomic study of the Phyllosoma complex and other triatomine (Insecta: Hemiptera: Reduviidae) species of epidemiological importance in the transmission of Chagas disease: using ITS-2 and mtCytB sequences. Mol. Phylogenet. Evol. 41 (2), 279-287.

2 Harry,M., Dupont,L., Quartier,M., Diotaiuti,L., Walter,A. and Romana,C. 2009. New perspectives for population genetics of Chagas'disease vectors in the Northeastern Brazil: isolation of polymorphic microsatellite markers in Triatoma brasiliensis. Infect. Genet. Evol. 9 (4), 633-637.

3 Waleckx,E., Salas,R., Huaman,N., Buitrago,R., Bosseno,M.F., Aliaga,C., Barnabe,C., Rodriguez,R., Zoveda,F., Monje,M., Baune,M., Quisberth,S., Villena,E., Kengne,P., Noireau,F. and Breniere,S.F. New insights on the Chagas disease main vector Triatoma infestans (Reduviidae, Triatominae) brought by the genetic analysis of Bolivian sylvatic populations. 2011. Infect. Genet. Evol. 11 (5), 1045-1057.

4 Harris,K.D. and Beard,C.B. Evidence of a species complex in the Chaga's vector Triatoma dimidiate. Unpublished

5 Dorn,P.L., Calderon,C., Melgar,S., Moguel,B., Solorzano,E., Dumonteil,E., Rodas,A., de la Rua,N., Garnica,R. and Monroy,C. 2009. Two Distinct Triatoma dimidiata (Latreille, 1811) Taxa Are Found in Sympatry in Guatemala and Mexico. PLoS Negl Trop Dis 3 (3), E393.

6 Giordano,R., Pizarro,J.C., Paulk,S. and Stevens,L. Genetic diversity of Triatoma infestans (Hemiptera: Reduviidae) in Chuquisaca, Bolivia based on the mitochondrial cytochrome b gene. Unpublished

7 Pfeiler,E., Bitler,B.G., Ramsey,J.M., Palacios-Cardiel,C. and, Markow,T.A. 2006. Genetic variation, population structure, and phylogenetic relationships of Triatoma rubida and T. recurva (Hemiptera: Reduviidae: Triatominae) from the Sonoran Desert, insect vectors of the Chagas' disease parasite Trypanosoma cruzi Mol. Phylogenet. Evol. 41 (1), 209-221.

8 Lyman,D.F., Monteiro,F.A., Escalante,A.A.,Cordon-Rosales,C., Wesson,D.M., Dujardin,J.P. and Beard,C.B. 1999. Mitochondrial DNA sequence variation among triatomine vectors of Chagas' disease. Am. J. Trop. Med. Hyg. 60 (3), 377-386.

9 Martinez-Hernandez,F., Martinez-Ibarra,J.A., Catala,S., Villalobos,G., de la Torre,P., Laclette,J.P., Alejandre-Aguilar,R., and Espinoza,B. 2010. Natural crossbreeding between sympatric species of the phyllosoma complex (Insecta: Hemiptera: Reduviidae) indicate the existence of only one species with morphologic and genetic variations. Am. J. Trop. Med. Hyg. 82 (1), 74-82.

10 Rocha,C.S., Silva,M.T.A., Ambrosio,D.L., Gardim,S., Cicarelli,R.M.B. and Rosa,J.A. Genetic Variability of Three Populations of Triatoma rubrovaria from Southern Brazil Evaluated by Mitochondrial DNA Sequences. Unpublished

11 Justi,S.A., Noireau,F., Cortez,M.R. and Monteiro,F.A. The sylvatic ecotope of Rhodnius stali in Alto Beni, Bolivia. Unpublished

12 Monteiro,F.A., Barrett,T.V., Fitzpatrick,S., Cordon-Rosales,C., Feliciangeli,D. and Beard,C.B. 2003. Molecular phylogeography of the Amazonian Chagas disease vectors Rhodnius prolixus and R. robustus. Mol. Ecol. 12 (4), 997-1006.

13 Garcia,B.A. and Powell,J.R. 1998. Phylogeny of species of Triatoma (Hemiptera: Reduviidae) based on mitochondrial DNA sequences. J. Med. Entomol. 35 (3), 232-238.

14 Piccinali,R.V., Marcet,P.L., Noireau,F., Kitron,U., Gurtler,R.E., and Dotson,E.M. 2009. Molecular population genetics and phylogeography of the Chagas disease vector Triatoma infestans in South America. J. Med. Entomol. 46 (4), 796-809.

15 Ceballos,L.A., Piccinali,R.V., Berkunsky,I., Kitron,U. and Gurtler,R.E. 2009. First finding of melanic sylvatic Triatoma infestans (Hemiptera: Reduviidae) colonies in the Argentine Chaco. J. Med. Entomol. 46 (5), 1195-1202.

16 Gaunt,M.W. and Miles,M.A. 2002. An insect molecular clock dates the origin of the insects and accords with palaeontological and biogeographic landmarks. Mol. Biol. Evol. 19 (5), 748-761.

17 Patterson,J.S. and Gaunt,M.W. 2010. Phylogenetic multi-locus codon models and molecular clocks reveal the monophyly of haematophagous reduviid bugs and their evolution at the formation of South America. Mol. Phylogenet. Evol. 56 (2), 608-621.

18 Calleros,L., Panzera,F., Bargues,M.D., Monteiro,F.A., Klisiowicz,D.R., Zuriaga,M.A., Mas-Coma,S. and Perez,R. 2010. Systematics of Mepraia (Hemiptera-Reduviidae): cytogenetic and molecular variation. Infect. Genet. Evol. 10 (2), 221-228.

19 Klass,K.-D., Picker,M.D., Damgaard,J., van Noort,S. and Tojo,K. 2003. The Taxonomy, Genitalic Morphology, and Phylogenetic Relationships of South African Mantophasmatodea (Insecta). Entomol. Abh (Dres). 61 (1), 3-67.

20 Sainz,A.C., Mauro,L.V., Moriyama,E.N. and Garcia,B.A. 2004. Phylogeny of triatomine vectors of Trypanosoma cruzi suggested by mitochondrial DNA sequences. Genetica 121 (3), 229-240.

21 Garcia,B.A., Moriyama,E.N. and Powell,J.R. 2001. Mitochondrial DNA sequences of triatomines (Hemiptera: Reduviidae): phylogenetic relationships. J. Med. Entomol. 38 (5), 675-683.

22 Garcia,B.A., Manfredi,C., Fichera,L. and Segura,E.L. 2003. Short report: variation in mitochondrial 12S and 16S ribosomal DNA sequences in natural populations of Triatoma infestans (Hemiptera: Reduviidae). Am. J. Trop. Med. Hyg. 68 (6), 692-694.

23 Hypsa,V., Tietz,D.F., Zrzavy,J., Rego,R.O., Galvao,C. and Jurberg,J. 2002. Phylogeny and biogeography of Triatominae (Hemiptera: Reduviidae): molecular evidence of a New World origin of the Asiatic clade. Mol. Phylogenet. Evol. 23 (3), 447-457.

24 Segura,E.L., Torres,A.G., Fusco,O. and Garcia,B.A. 2009. Mitochondrial 16S DNA variation in populations of Triatoma infestans from Argentina. Med. Vet. Entomol. 23 (1), 34-40.

25 Weirauch,C. and Munro,J.B. 2009. Molecular phylogeny of the assassin bugs (Hemiptera: Reduviidae), based on mitochondrial and nuclear ribosomal genes. Mol. Phylogenet. Evol. 53 (1), 287-299.

26 Stothard,J.R., Yamamoto,Y., Cherchi,A., Garcia,A.L., Valente,S.A.S., Schofield,C.J. and Miles,M.A. 1998. A preliminary survey of mitochondrial sequence variation within triatomine bugs (Hemiptera:Reduviidae) using single strand conformational polymorphism (SSCP) analysis and direct sequencing. Bull. Entomol. Res. 88 (5), 553-560 (1998)

27 Vendrami,D.P., Ceretti,W. Jr. and Marrelli,M.T. Taxonomic and systematic analysis among triatomine species (Hemiptera: Reduviidae) of colonies from SESA (Special Health Service of Araraquara), inferred from 16S mitochondrial DNA sequences. Rev Bras Entomol. In press

28 Marcilla,A., Bargues,M., Ramsey,J., Dujardin,J., Schofield,C. and Mas-Coma,S. Primary and secondary structures of the 18S rRNA gene and phylogenetic analysis of Triatominae (Hemiptera: Reduviidae), vectors of Chagas disease. Unpublished

29 Bargues,M.D., Klisiowicz,D.R., Gonzalez-Candelas,F., Ramsey,J.M. Monroy,C., Ponce,C., Salazar-Schettino,P.M., Panzera,F., Abad-Franch,F., Sousa,O.E., Schofield,C.J., Dujardin,J.P., Guhl,F. 2008. and Mas-Coma,S. 2008. Phylogeography and genetic variation of Triatoma dimidiata, the main Chagas disease vector in Central America, and its position within the genus Triatoma. PLoS Negl Trop Dis 2 (5), E233.

30 Dorn,P.L., Calderon,C., Melgar,S., Moguel,B., Solorzano,E. Dumonteil,E., Rodas,A., de la Rua,N., Garnica,R. and Monroy,C. 2009. Two Distinct Triatoma dimidiata (Latreille, 1811) Taxa Are Found in Sympatry in Guatemala and Mexico. PLoS Negl Trop Dis 3 (3), E393.

31 Marcilla,A., Bargues,M.D., Ramsey,J.M., Magallon-Gastelum,E., Salazar-Schettino,P.M., Abad-Franch,F., Dujardin,J.P., Schofield,C.J. and Mas-Coma,S. 2001. The ITS-2 of the nuclear rDNA as a molecular marker for populations, species, and phylogenetic relationships in Triatominae (Hemiptera: Reduviidae), vectors of Chagas disease. Mol. Phylogenet. Evol. 18 (1), 136-142.

32 Villalobos,G., Martinez-Hernandez,F., de la Torre,P., Laclette,J.P. and Espinoza,B. 2011. Entomological Indices, Feeding Sources, and Molecular Identification of Triatoma phyllosoma (Hemiptera: Reduviidae), One of the Main Vectors of Chagas Disease in the Istmo de Tehuantepec, Oaxaca, Mexico. Am. J. Trop. Med. Hyg. 85 (3), 490-497.

33 Martinez-Hernandez,F., Martinez-Ibarra,J.A., Villalobos,G., De la Torre,P., Laclette,J.P. and Espinoza,B. Genetic variation of North American Triatomines (Insecta: Hemiptera: Reduviidae): initial divergence between species and populations of Chagas disease vector. Unpublished

34 Marcilla,A., Bargues,M.D., Abad-Franch,F., Panzera,F., Carcavallo,R.U., Noireau,F., Galvao,C., Jurberg,J., Miles,M.A., Dujardin,J.P. and Mas-Coma,S. 2002. Nuclear rDNA ITS-2 sequences reveal polyphyly of Panstrongylus species (Hemiptera: Reduviidae: Triatominae), vectors of Trypanosoma cruzi. Infect. Genet. Evol. 1 (3), 225-235.
